# Surface Treatments for Enhancing the Bonding Strength of Aluminum Alloy Joints

**DOI:** 10.3390/ma16165674

**Published:** 2023-08-18

**Authors:** Juncheng Luo, Jianhua Liu, Huanxiong Xia, Xiaohui Ao, Haojie Yin, Lei Guo

**Affiliations:** 1 School of Mechanical Engineering, Beijing Institute of Technology, Beijing 100081, China; 18801107308@163.com (J.L.); jeffliu@bit.edu.cn (J.L.); xhao@bit.edu.cn (X.A.);; 2 Tangshan Research Institute, Beijing Institute of Technology, Tangshan 063015, China; 3 Air Force Medical Center, PLA, Beijing 100142, China

**Keywords:** surface treatment, laser ablation, phosphoric acid anodizing, microstructure, bonding strength

## Abstract

Aluminum alloy adhesive bonding joint widely appears in many industrial products. Improving the mechanical performances of aluminum alloy bonding joints has been attracting much effort. To acquire more excellent bonding strength, this paper focused on the effects of different surface treatments, including laser ablation and milling superposed by phosphoric acid anodizing (PAA). The treated surfaces were characterized by roughness and contact angle, and the effects of the geometric parameters of microstructures on wettability, failure mode, and shear strength were examined. The results indicate that those surfaces where the spacing is smaller than the diameter present a hydrophilic property and the corresponding specimens are mainly subject to cohesive failure, and vice versa. Additionally, laser ablation with a properly designed dimple pattern can greatly improve the bonding strength, and the maximum average shear strength of specimens with a thickness of 50 μm reaches 32.82 MPa, which is an increase of 28.15% compared with the original milling specimen. Moreover, fabricating groove or grid patterns on the surfaces and applying PAA treatment can also significantly enhance the bonding strength, reaching up to 36.28 MPa.

## 1. Introduction

Adhesive bonding joint is increasingly applied in automobile [1], marine [2], and aerospace [3,4] industrial products due to the distinct advantages of simple structure, small size, and uniform stress distribution. Aluminum alloy widely appears in adhesive bonding joints owing to its lightweight, high specific strength, and good corrosion resistance [1,5]. However, aluminum alloy joints are usually involved in failure problems, especially in some precision components [6,7], and thus improving the bonding strength needs continual effort.

The bonding performances of adhesive bonding joints including strength [2], fatigue [8], aging [9], and fracture [6], all depend on the mechanical and physicochemical characteristics of adhesives and substrates, in particular summarizing five aspects [10]: (i) material properties of adhesives and substrates; (ii) technological methods like surface treatment to the substrates and curing conditions of the adhesives; (iii) geometrical parameters such as overlap width, bondline thickness, etc.; (iv) loading manners; and (v) environmental conditions, such as temperature and humidity. To acquire excellent adhesive bonding performance, engineers designed various surface treatments to modify the surface topography and improve the surface wettability [11,12].

In general, surface treatment technologies of aluminum alloy include machining, sandblasting, chemical etching, anodizing, silane, laser ablation, etc. Machining and sandblasting are mainly able to increase the surface roughness of substrates to enhance mechanical interlocking with the purpose to improve bonding strength [13,14], despite the phenomenon that a few abrasives may be impacted into the substrate to vary chemical characteristics [15]. Whereas some surface treatment technologies, including chemical etching, anodizing, and plasma ablation, are mainly subject to improving wettability to enhance bonding strength [11,16,17,18]. Compared with other technologies, laser ablation has the power to increase surface roughness and improve wettability to enhance bonding strength, with the characteristics of friendly environment, repeatable processing, easy industrialization, and low maintenance cost [19,20]. The principle of laser ablation is to utilize the high energy of pulses to heat the radiated areas and to form a plasma plume moving outwards through the melting and vaporizing process, and hence a melt cavity with microcracks or micropores is fabricated after the laser beam leaves, while the plasma plume will finally congeal and fall back in surrounding areas of the melt cavity [19].

The effects of laser ablation on adhesive bonding have been reported from many aspects. Wong et al. [21] explained the formation mechanisms of the melt cavity and microcrack on the aluminum alloy surface with laser ablation and claimed that the bonding performance can be improved by adjusting the laser parameters. Critchlow et al. [22,23] found that laser ablation can remove the organic contaminants on the substrate surface to enhance surface chemical energy and then the shear strength with an increase of 22% compared with degreasing treatment. Besides, the effect of laser ablation was equivalent to phosphoric acid anodizing (PAA) in terms of enhancing bonding performance, while laser ablation treatment is more environmentally friendly [24]. Afterward, Baburaj et al. [25] identified the internal mechanisms of laser ablation on enhancing bonding performance, including (i) an increase in the actual contact area, (ii) the mechanical locking of adhesive between microstructures, and (iii) an improvement in surface wettability. Subsequently, Alfano et al. [26] discovered a phenomenon that the adhesive did not perfectly fill the melt cavity fabricated with laser ablation, which indicated that a better bonding performance can be obtained by improving the filling effect of the adhesive. Lately, abundant studies involving technological parameters of laser ablation were conducted. The effects of laser power, scanning speed, and radial hatching distance on the bonding strength of butt joints were studied. Romoli et al. [27] found that the maximum bonding strength was approximately 30% higher than the as-received bonding strength. In addition, when surface roughness exceeded a constant value, the higher roughness was, the more likely the air was stuck in the recesses of melt cavities, which could harm the bonding performances. The effects of scanning speed, pulse frequency, and hatching distance on bonding performance and wettability were examined using a Box–Behnken three-level factorial design, and the bonding performances of the aluminum alloy joints were enhanced using properly selected laser parameters [28]. Zhu et al. [29] ulteriorly studied the effects of laser power on the bonding performance of aluminum alloy formed by sheet, extrusion, and cast, respectively. Several similar works referring to the effects of laser parameters on shear strength [30,31,32,33] and fracture behavior [34,35] claimed that laser ablation could significantly affect wettability and bonding performance. Prakash [34] emphasized that the dimensions of dimples including texture depth and pitch were the key factors to the bonding strength, but the effect of the texture was not examined.

Some works around the technological parameters of laser ablation have been performed, while more complicated issues on geometrical parameters of surface textures fabricated by laser ablation need to be clarified. Additionally, the comparison between the laser ablation and other surface treatments such as micro-pattern and phosphoric acid anodizing (PAA) also need to be clarified. This study focuses on the effects of different surface treatments, including laser ablation, micro-pattern, and phosphoric acid anodizing (PAA), on the shear strength of aluminum alloy single-lap joints, where the laser ablation is applied to fabricate designed microstructures on the aluminum alloy substrate. The morphology, microstructures, contact angle, failure modes, and bonding strength of the treated surfaces are examined in various aspects. This work would help understand the bonding mechanisms of different surface treatments and provide a way to enhance the bonding strength of aluminum alloy joints.

## 2. Materials and Methods

### 2.1. Materials

Lightweight hard aluminum alloy, 2A12-T4 (GB/T 3191-1998), with a high tensile strength of over 400 MPa and elasticity modulus of 70 GPa was selected as the substrate of all adhesive bonding joints. A three-component system based on diglycidyl ether of bisphenol A (DGEBA), nitrile rubber (CTBN), and 2-ethyl-4-methylimidazole (EM 2, 4) at a weight ratio of 100:35:8 was applied as the adhesive. The curing conditions of the adhesive were suggested for 4 h at 80 °C. And the glass transition temperature (*T_g_*) was proved to be approximately 115 °C by a standard Differential Scanning Calorimetry (DSC) test.

### 2.2. Surface Treatments and Specimen Preparation

**Figure 1:**
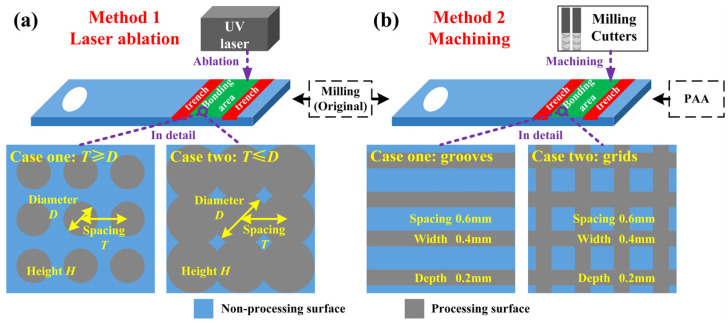
Schematics of the substrates with different surface features: (**a**) dimple-pattern fabricated by laser ablation and (**b**) groove- or grid-pattern fabricated by machining.

The original substrate surface was produced by precision milling. Then, surface treatments, including laser ablation, phosphoric acid anodizing (PAA), and machining, were used to modify the surface characteristics of the aluminum alloy substrates to enhance the bonding strength. Specifically, a micro-dimple machining system (Fermi Laser, Shanghai, China), including UV laser and measurement devices was used to fabricate cyclical dimples on the surface of the substrates, as shown in Figure 1a. The process parameters of the laser beam in the micro-dimple machining system are summarized in Table 1. Three parameters, including spacing between the adjoining dimples *T*, dimple height *H*, and dimple diameter *D*, are used to define the geometry of the pattern. Seven groups of substrates with dimple-pattern were designed and fabricated by laser ablation, and four groups of substrates with groove- or grid-pattern were fabricated by a CNC milling machine on either the original surfaces or those treated by PAA, as shown in Figure 1b. Additionally, the original substrate and that treated by PAA only were prepared as two control groups. Therefore, thirteen groups of substrates with different surface features were prepared, and more details are listed in Table 2. Notably, the substrates were marked to distinguish their surface features, where specimens 1–7 represented the substrates with dimple patterns fabricated by laser ablation, specimens 8–9 were treated by machining, and specimens 10–11 were treated by PAA and machining.

**Figure 2:**
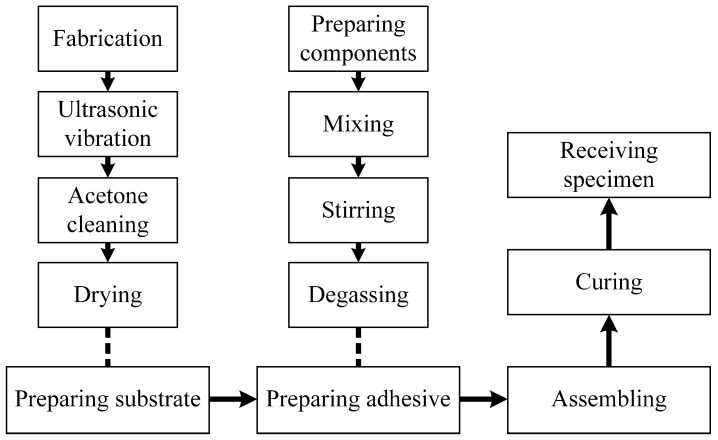
A flowchart of preparing a SLJ specimen.

**Figure 3:**
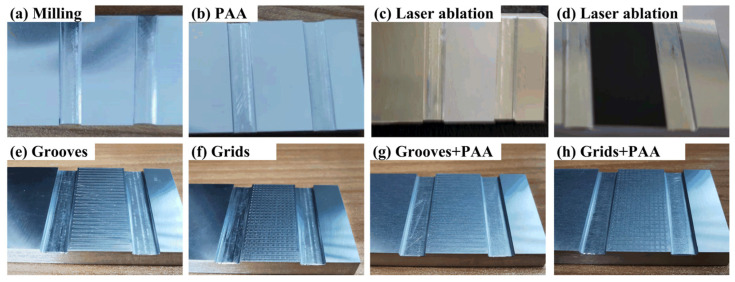
Substrates with different surface treatments.

**Figure 4:**
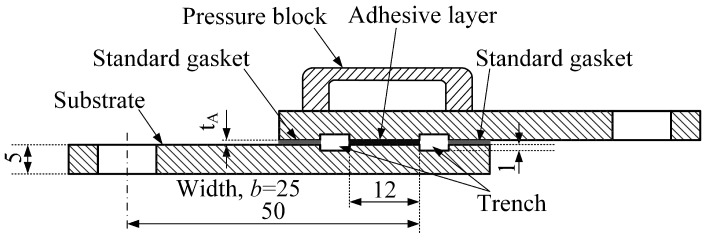
Principle and key parameters of the tooling for assembling SLJ specimens, where *t_A_* is the adhesive thickness.

**Figure 5:**
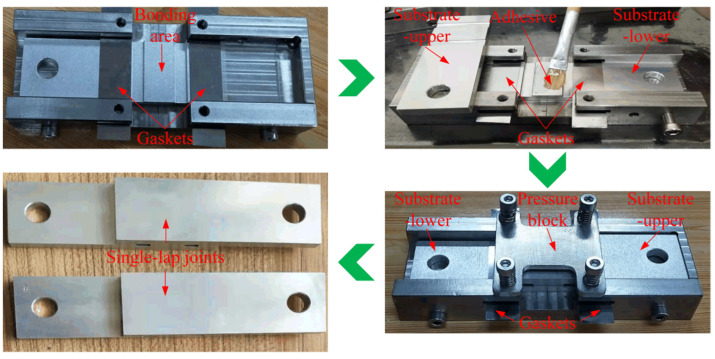
Making procedure of a SLJ specimen.

A flowchart describing the process of receiving a SLJ specimen is presented in Figure 2. It includes five steps: preparing substrates, preparing adhesive, assembling, curing, and receiving specimens. Additionally, both preparing substrates and preparing adhesive include four substeps. The substrates treated by different surface treatments are presented in Figure 3. A distinct difference in the shade for laser ablation with different parameters can be seen in Figure 3c,d. Subsequently, all the substrates were treated for 10 min by ultrasonic vibration to remove physical impurities, such as metal debris, dust, etc. Besides, acetone cleaning was repeatedly applied to remove surface chemical pollutants, including grease and sediment. Finally, all the substrates were dried. As for preparing adhesive, three components were first weighed by a high-precision electronic scale and then mixed and stirred for 10 min. Finally, a vacuum degassing method was conducted to remove the air bubbles stuck in the adhesive. In the assembling process, a special tooling referring to ASTM D1002 [36] was made to assemble the SLJ specimens. The principle and key parameters of the tooling are shown in Figure 4, where two standard gaskets are applied to precisely control the bondline thickness under the constraint force from the pressure block. Additionally, the size of the bonding area is 12 mm × 25 mm, and two trenches on both sides of the adhesive layer were fabricated to remove the adhesive fillet. Figure 5 shows the actual assembling procedure of the SLJ specimen, the standard gasket (Fein Tool, Shenzhen, China) with 50 μm thickness was used to control the bondline thickness. After the assembling module, the assembled specimens were put in a thermostat to cure for 4 h at 80 °C and then naturally cooled in the thermostat. Lastly, the tooling was disassembled and the SLJ specimens were received. The process of receiving SLJ specimens was also applied in our previous works [6,11].

### 2.3. Testing Methods and Devices

A scanning electron microscope (Regulus 8230, Hitachi Co., Ltd., Tokyo, Japan) and a white light interferometer (Contour GT-K, Bruker Co., Berlin, Germany) were used to observe the morphology of the substrate surfaces. With the aid of the software VISION64, roughness Ra was evaluated. At least three different areas were tested per each specimen and the average Ra was calculated.

A contact angle meter (JC2000C1, Shanghai Zhongchen Digital Technology Equipment Co., Ltd., Shanghai, China) was utilized to measure contact angle (CA). Ultrapure water of 2 μL was dropped gently on the surfaces to evaluate their wettability. Since the water drop moves on the surface with microstructures, 500 photos were captured in 20 s. For the specimens on which the water drop presented a stable shape, the CA was then calculated. Similarly, at least three different areas were tested per each specimen and the average CA was calculated to characterize wettability.

**Figure 6:**
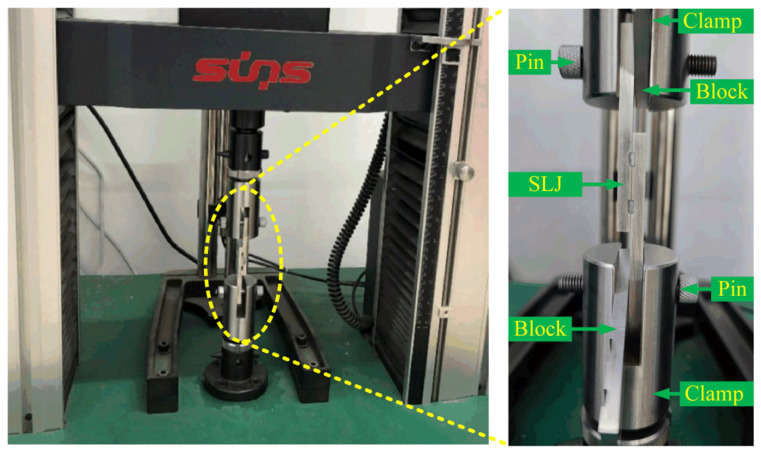
Shear tests for the SLJs.

An electronic material testing machine (UTM4304, Sansi Zongheng Technology Co., Ltd., Shenzhen, China) was utilized to obtain the shear strength of the SLJ specimens, as shown in Figure 6. The tensile rate was set to 1 mm/min [37,38,39], the ambient temperature was 26 ± 3 °C, and the relative humidity was less than 70%. At least three tests were conducted, and the average shear strength was calculated.

## 3. Results and Discussion

### 3.1. Surface Morphology and Roughness

**Figure 7:**
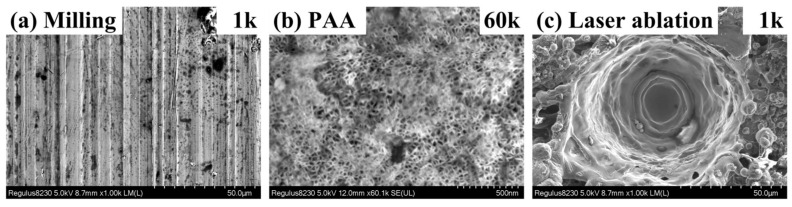
Surface morphologies obtained by SEM: (**a**) milling-1k; (**b**) PAA-60k; (**c**) laser ablation-1k.

Figure 7 shows the morphologies of the substrate surfaces with milling, PAA, and laser ablation, respectively, in the microscale or nanoscale obtained by SEM. The surfaces fabricated by milling have a characteristic of wavy morphology, as shown in Figure 7a, which results from the reciprocating cutting of milling cutters. After laser ablation, the surface presents a rugged morphology with preconceived melt cavities and surrounding coagula, as shown in Figure 7c. The surface treated by PAA demonstrates a porous morphology with nanoscale structural features, as shown in Figure 7b.

**Figure 8:**
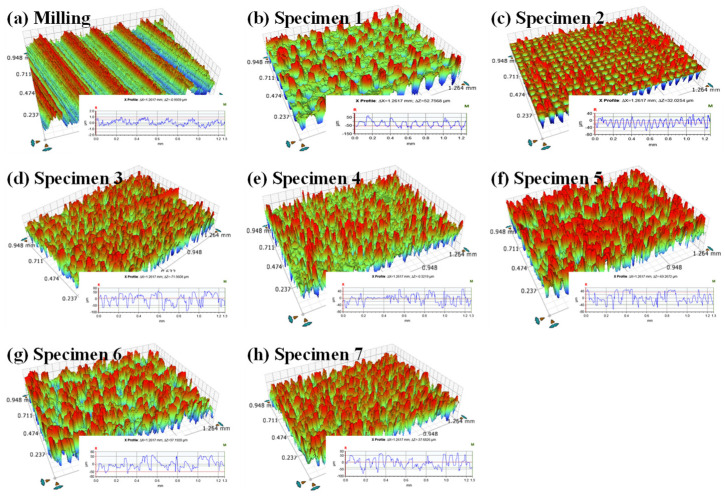
3D morphologies and 2D profiles of original milling surface and surfaces consisting of cyclical dimples fabricated by laser ablation. A rainbow spectrum was applied to describe 3D morphologies: the red and the blue represent the peak and the valley, respectively.

The 3D morphologies and 2D profiles of the original milling surface and the surfaces consisting of cyclical dimples fabricated by laser ablation are presented in Figure 8. It can be seen that the original milling surface presents an obvious wave-texture feature, as shown in Figure 8a, and the surfaces of specimen 1 and specimen 2 present a dimple-texture feature, as shown in Figure 8b,c. The other morphologies are rugged and irregular, as seen in Figure 8d-h. Two reasons account for the aforementioned features. One is the size relationship between the spacing *T* and the diameter *D*. When the value of *T* is greater than *D*, the dimples are unbroken. Otherwise, the dimples are unclear in the images. The other results from the fact that the melted aluminum alloy moves outwards, and then the vaporized aluminum alloy substrate congeals fall on the surrounding area [19].

Ra, the arithmetic mean deviation of the assessed profiles, is calculated to quantify the surface roughness of the substrates. Table 3 lists the Ra of the original milling surface and the laser-ablation surfaces. Compared with the original milling surface, the Ra of the laser-ablation surfaces significantly increased. This indicates that laser ablation reshaped the original milling surface. For the surfaces treated by laser ablation, the denser the dimples are, the larger Ra is. This is because more aluminum alloy congeals pile up on unabated areas to form higher peaks.

### 3.2. Contact Angle

**Figure 9:**
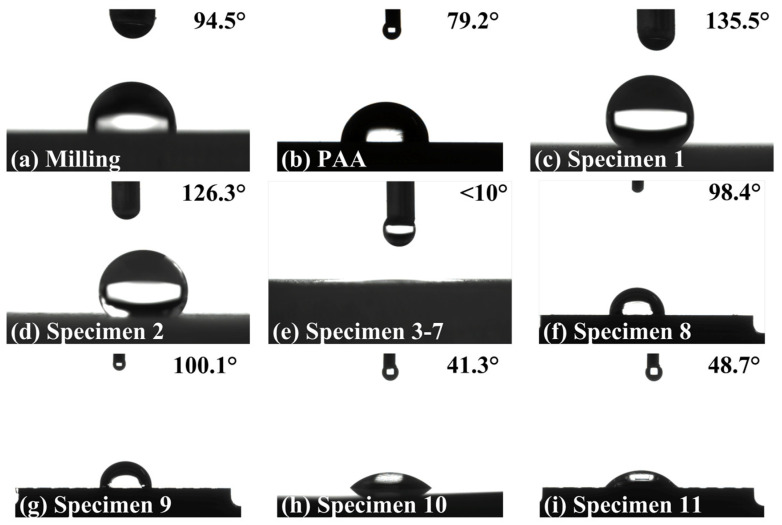
Contact angles of the substrates with different surface treatments.

**Figure A1:**
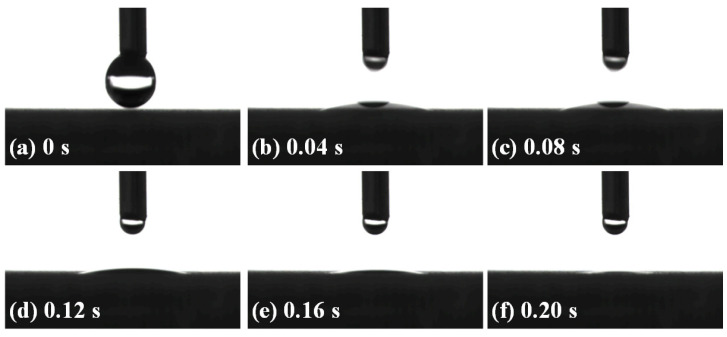
A typical dynamic process where ultrapure water drops on the surfaces of specimens 3–7.

To examine the wettability of different surfaces treated by milling, PAA, laser ablation, and machining, contact angle (CA) tests were conducted, and the corresponding results were obtained and shown in Figure 9. The CA of the milling surface is about 94.5°, representing weakly hydrophobic, while the CA of the PAA surface is about 79.2°, representing slightly hydrophilic, as shown in Figure 9a,b. As for laser ablation, the surfaces of specimen 1 and specimen 2 represent worse wettability, and their CAs increase to 135.5° and 126.3° compared with the milling surface, respectively, as shown in Figure 9c,d. We noted that ultrapure water infiltrated into dimples on the surfaces of specimens 3–7, differing from a stable shape on the surfaces of specimens 1–2. The evidence captured by a continuous shooting camera is shown in Figure A1. Consequently, the CAs of these surfaces are considered to be less than 10°, as shown in Figure 9e.

In general, a meaningful CA can be calculated once a stable state is presented on the tested surfaces. And the stable state depends on three kinds of interactions, including the Laplace pressure difference generated from the unique geometric structure, the gravity of ultrapure water, and atmospheric pressure difference [40]. The gravity of ultrapure water is approximately constant. However, as the ultrapure water continually infiltrates into the tested surface, the three-phase contact state changes, resulting in the change of Laplace pressure difference, but the Laplace pressure difference cannot prevent the ultrapure water from moving downward due to the gravity force. As for atmospheric pressure difference, it is considered case-by-case and its direction is always upward. For the case where *T* is larger than *D*, the adjacent dimples on the surfaces of specimen 1 and specimen 2 are separate and blind. Consequently, the atmospheric pressure difference becomes larger and larger during the infiltration process so that a three-phase equilibrium will be achieved before the ultrapure water reaches the bottom of the dimples. This is the reason why the drops on the surfaces of specimen 1 and specimen 2 can maintain a stable shape. However, as for specimens 3–7, the atmospheric pressure difference approximately equals zero due to the air in the dimples connecting with the atmosphere. With the interactions of the Laplace pressure difference and the gravity of ultrapure water, the drops can wet the bottom of dimples and then spread over the tested surface, which presents a visible infiltration phenomenon during the CA tests.

As shown in Figure 9f,g, the CAs of groove- and grid-pattern surfaces without PAA are about 98.4° and 100.1°, respectively, closing to the result of the milling surface and representing a slightly hydrophobic property. By contrast, the hydrophilic property appears on the groove- and grid-pattern surfaces with PAA treatment, and their CAs are about 41.3° and 48.7°, respectively, as shown in Figure 9h,i. Additionally, a distinct decrease of CAs occurs once the groove- or grid-pattern is fabricated on the PAA substrates compared with the milling substrate (no PAA), and the decrement is much larger than the difference between the PAA and the milling surfaces. However, the difference in CAs is slight between the groove-pattern and the grid-pattern surfaces, whether the PAA treatment is applied or not.

### 3.3. Strength Analysis

The shear strengths and their fluctuations of the SLJs with different surface treatments are listed in Table 4. The average shear strengths of specimens treated by milling and PAA are 25.61 MPa and 33.45 MPa, respectively, while the shear strength increases to 34.68 MPa (Specimen 7) for the specimens treated by laser ablation, and the increment is about 35.42% compared with the milling (25.61MPa). The maximum shear strength is given by the laser-ablation treatment at the dimple pattern with the geometric parameters: *T* = 30 μm, *H* = 45 μm, and *D* = 70 μm. However, the minimum shear strength of the specimens treated by laser ablation is about 23.97 MPa (Specimen 1) obtained at the conditions: *T =* 120 μm, *H* = 30 μm, *D =* 60 μm, which even decreases 6.40% more than the milling specimen. Therefore, the shear strength of the laser-ablation surfaces greatly depends on the geometric parameters of the dimple pattern.

**Figure 10:**
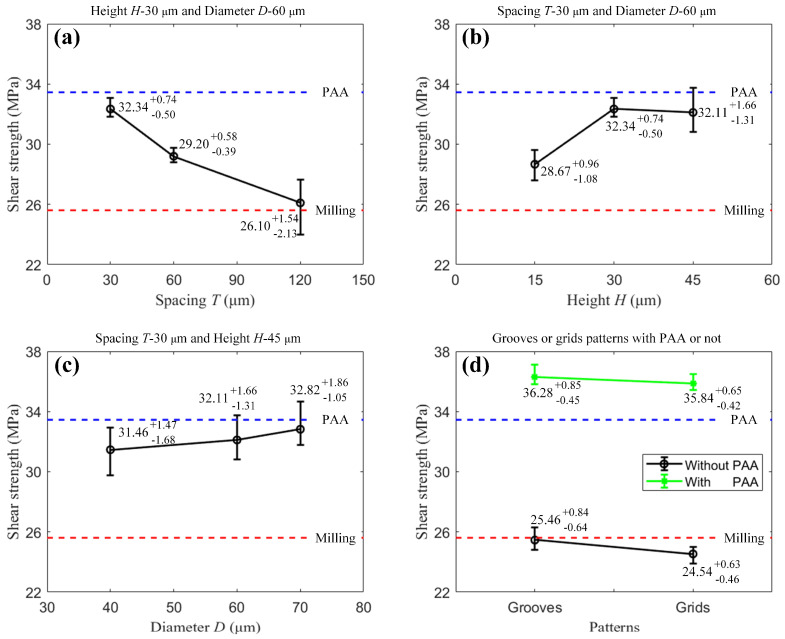
Shear strengths of the specimens with different microstructures and treatments: (**a**) spacing, (**b**) height, (**c**) diameter, and (**d**) patterns.

To examine the effect of spacing *T* on the shear strength of the laser-ablation specimens, we compare specimens 1, 2, and 3, where the height *H* is 30 μm, the diameter *D* is 60 μm, and the spacings *T* are 120 μm, 60 μm, and 30 μm, respectively. As shown in Figure 10a, the average shear strengths of specimens 1, 2, and 3 are about 26.10 MPa, 29.20 MPa, and 32.34 MPa, respectively. This indicates that the shear strength increases as the spacing decreases, and thus the shear strength is enhanced with denser dimples fabricated on the substrate. Compared with the PAA and milling specimens, the shear strength at a spacing of 30 μm is close to the level of the specimen treated by PAA, while the shear strength at a spacing of 120 μm decreases to the level of the original milling specimen.

To examine the effects of height *H* and diameter *D* on the shear strength of the laser-ablation specimens, we compare specimens 3, 4, and 5 for height *H*, and specimens 5, 6, and 7 for diameter *D*. As shown in Figure 10b, the average shear strengths are 28.67 MPa, 32.34 MPa, and 32.11 MPa at the height of 15 μm, 30 μm, and 45 μm, respectively. It can be seen that the shear strength increases first and then decreases slightly as the height increases. Figure 10c shows that the average shear strengths are 31.46 MPa, 32.11 MPa, and 32.82 MPa at the diameter of 40 μm, 60 μm, and 70 μm. This indicates that the shear strength is insensitive to the diameter in the range of 40–70 μm at the height of 45 μm and the spacing of 30 μm.

To examine the effects of the groove and the grid patterns with PAA treatment, we compare specimens 8 and 9, and 10 and 11. Figure 10d shows that the average shear strengths of the groove- and grid-pattern specimens without PAA treatment are 25.46 MPa and 24.54 MPa, respectively, near the shear strength of 25.61 MPa of the original milling specimen, while the corresponding strengths are 36.28 MPa and 35.84 MPa with PAA treatment. It can be seen that PAA treatment can greatly improve the shear strength of the groove-pattern and the grid-pattern specimens, and the increments are significantly larger than that of the original milling specimen treated by PAA. Additionally, the difference is slight between the groove-pattern and the grid-pattern specimens, whether PAA treatment is applied or not. Furthermore, considering the surface wettability, we found that adding microstructures to the hydrophilic surfaces, such as the surfaces treated by PAA, can enhance the bonding strength.

The effects of different geometric parameters (spacing: 120 μm, 60 μm, and 30 μm; height: 15 μm, 30 μm, and 45 μm; diameter: 40 μm, 60 μm, and 70 μm), and patterns with PAA treatment or not on the shear strength were analyzed by using the ANOVA method. The spacing, height, diameter, PAA (or not), and patterns are factors A, B, C, D, and E, respectively, and the shear strength of the SLJ specimen is the dependent variable. The ANOVA results are listed in Table 5, showing that P_A_ = 0.0021 < 0.01, P_B_ = 0.0207 < 0.05, P_C_ = 0.6282 > 0.05, P_D_ = 0 < 0.01, P_E_ = 0.1141 > 0.05, and P_D×E_ = 0.5489 > 0.05. These results indicate that both spacing and PAA (or not) have extremely significant effects on the shear strength, and height has statistically significant effects on the shear strength. Additionally, there is no evidence to say that diameter, pattern, and the interaction between PAA (or not) and patterns have effects on the shear strength.

### 3.4. Failure Analysis

**Figure 11:**
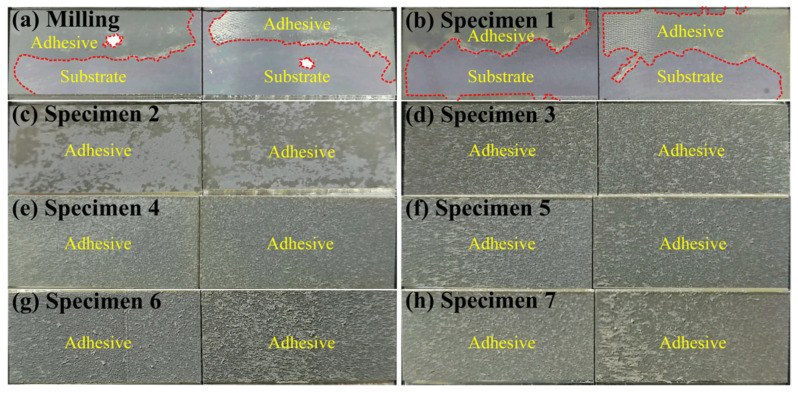
Fractured adhesive layers of specimens treated by milling and laser ablation.

The failure mode can be identified from the residual adhesive on the fractured bonding interface. The residual adhesives and substrate surfaces are marked for all the specimens and shown in Figure 11. The profiles of the residual adhesive are clear and are marked by red dotted lines for the milling surface and specimen 1, as shown in Figure 11a,b. The areas labeled as substrate are quite clean and almost half and half on the milling surface and specimen 1, and their average shear strengths are at a relatively low level with 25.61 MPa and 26.10 MPa, respectively. Therefore, it is a conclusion that the failure mode is mainly subject to interface failure. As for specimen 2, the residual adhesive occupies most of the bonding area and shows many spots, and a distinct difference in the shade can be seen in Figure 11c. The average shear strength of specimen 2 is 29.20 MPa, which is larger than milling and specimen 1. These indicate that the failure mode of specimen 2 is mainly subject to mixed failure with advantaged cohesive failure. As for the other specimens, the residual adhesives are spread over the bonding area of the substrates, as shown in Figure 11d–h. All surfaces of specimens 3–7 represent a strongly hydrophilic property, resulting in the adhesives infiltrating into the bottom of dimples. Adhesives, especially those infiltrated into the bottom of dimples, might be separated from the interior instead of the interface as the specimens were cracking. Additionally, the average shear strengths of specimens 3–7 are at a relatively high level. Therefore, we can conclude that the failure modes of specimens 3–7 are mainly subject to cohesive failure.

## 4. Conclusions

This study examined the effects of laser ablation, machining, and phosphoric acid anodizing (PAA), on the shear strength of aluminum alloy single-lap joints, and particular attention was paid to the microstructures fabricated by laser ablation. A series of testing experiments for morphology, wettability, and shear strength were carried out, and significance analysis and failure analysis were conducted. The findings are listed as follows:

(1) Laser ablation can reshape the original milling surface and greatly increase the Ra. However, it is not always conducive to improving surface wettability and bonding performance, depending on the geometric parameters of the dimple pattern;
(2) The size relationship between the spacing and diameter of the dimples significantly affects the CA and failure mode. Those surfaces where the spacing is smaller than the diameter present a hydrophilic property, and the failure modes of corresponding specimens are mainly subject to cohesive failure and vice versa;
(3) Geometric parameters, such as spacing and height of the dimples, greatly affect the bonding strength, while the diameter does not. And the maximum average shear strength of the specimens treated by laser ablation is about 32.82 MPa (at 30 μm spacing, 45 μm height, and 70 μm diameter), which is 28.15% higher than the original milling surface and approximately equivalent to the specimen treated by PAA;
(4) Patterns can significantly affect CA and shear strength, in particular, combining with PAA surface. However, there is no distinct difference between groove pattern and grid pattern in CA and shear strength, whether PAA treatment is applied or not;
(5) Microstructures fabricated by machining on a hydrophilic surface, such as the PAA surface, may significantly enhance the bonding strength, reaching up to 36.28 MPa. But the effect is slightly positive or even negative on a hydrophobic surface, like the milling surface, and the corresponding shear strength can reach down to 24.54 MPa, which is less than that of the milling specimen.

## Figures and Tables

**Table 1:** Process parameters of the laser beam.

Name of Parameters	Values
Power (W)	50
Wavelength (nm)	1064
Spot diameter (μm)	30
Focal distance (mm)	160

**Table 2:** Preparation methods and parameters of the substrates with different surface treatments.

Nature of Groups	Treatment Methods	Parameters
Control groups	Original (Milling)	Plane milling, flatness tolerance ≤ 0.015 mm.
	PAA	120–160 g/L phosphoric acid solution with a voltage 10 ± 0.5 V (DC) for 20 ± 1 min at 20 ± 5 °C solution temperature.
Test groups	Laser ablation	Spacing *T*, height *H*, and diameter *D* Specimen 1: *T* 120 μm, *H* 30 μm, *D* 60 μm; Specimen 2: *T* 60 μm, *H* 30 μm, *D* 60 μm; Specimen 3: *T* 30 μm, *H* 30 μm, *D* 60 μm; Specimen 4: *T* 30 μm, *H* 15 μm, *D* 60 μm; Specimen 5: *T* 30 μm, *H* 45 μm, *D* 60 μm; Specimen 6: *T* 30 μm, *H* 45 μm, *D* 45 μm; Specimen 7: *T* 30 μm, *H* 45 μm, *D* 70 μm.
	Machining	Specimen 8: Groove pattern with 0.6 mm spacing, 0.4 mm width, and 0.2 mm depth.
		Specimen 9: Grid pattern with 0.6 mm spacing, 0.4 mm width, and 0.2 mm depth.
	PAA and machining	Specimen 10: 120–160 g/L phosphoric acid solution with a voltage 10 ± 0.5 V (DC) for 20 ± 1 min at 20 ± 5 °C solution temperature, fabricated a groove pattern with 0.6 mm spacing, 0.4 mm width, and 0.2 mm depth.
		Specimen 11: 120–160 g/L phosphoric acid solution with a voltage 10 ± 0.5 V (DC) for 20 ± 1 min at 20 ± 5 °C solution temperature, fabricated a grid pattern with 0.6 mm spacing, 0.4 mm width, and 0.2 mm depth.

**Table 3:** Ra of the original milling surface and the laser-ablation surfaces.

Treatments	Specimen Numbers	Ra (μm)
Milling	No definition	0.352 ± 0.014
Laser ablation	1	11.566 ± 0.596
	2	19.799 ± 0.340
	3	27.829 ± 0.723
	4	17.025 ± 0.886
	5	25.785 ± 1.912
	6	26.574 ± 0.322
	7	26.959 ± 0.500

**Table 4:** The average shear strengths and fluctuations of specimens with different surface treatments.

Treatments		Average Shear Strength (MPa)	Fluctuation (MPa)	
			Downward	Upward
Milling		25.61	−0.79	+0.96
PAA		33.45	−0.42	+0.56
Laser ablation	Specimen 1	26.10	−2.13	+1.54
	Specimen 2	29.20	−0.39	+0.58
	Specimen 3	32.34	−0.50	+0.74
	Specimen 4	28.67	−1.08	+0.96
	Specimen 5	32.11	−1.31	+1.66
	Specimen 6	31.46	−1.68	+1.47
	Specimen 7	32.82	−1.05	+1.86
Machining	Specimen 8	25.46	−0.64	+0.84
	Specimen 9	24.54	−0.63	+0.46
PAA and machining	Specimen 10	36.28	−0.45	+0.85
	Specimen 11	35.84	−0.42	+0.65

**Table 5:** The ANOVA results for the shear strength of SLJs.

Source	SS	DF	MS	F Value	*p* Value
Factor A: spacing	58.41	2	29.20	20.29	0.0021
Error	8.64	6	1.44		
Total	67.04	8			
Factor B: height	22.98	2	11.49	7.92	0.0207
Error	8.70	6	1.45		
Total	31.68	8			
Factor C: diameter	3.04	2	1.52	0.5	0.6282
Error	18.13	6	3.02		
Total	21.17	8			
Factor D: PAA (or not)	366.97	1	366.97	831.8	0
Factor E: pattern	1.39	1	1.39	3.14	0.1141
Interaction D×E	0.17	1	0.17	0.39	0.5489
Error	3.53	8	0.44		
Total	372.06	11			

## Data Availability

Not applicable.

## Appendix A

As for specimens 3–7, where the spacing *T* is smaller than the diameter *D*, ultrapure water infiltrated into the dimples instead of keeping a stable shape on the substrate surfaces. Consequently, ultrapure water almost disappears after 0.20 s (the time will be started when ultrapure water is dropping). The frames captured by a continuous shooting camera at 25 fps are shown in Figure A1.
